# Two novel deep-sea sediment metagenome-derived esterases: residue 199 is the determinant of substrate specificity and preference

**DOI:** 10.1186/s12934-018-0864-4

**Published:** 2018-01-30

**Authors:** Ying-Yi Huo, Shu-Ling Jian, Hong Cheng, Zhen Rong, Heng-Lin Cui, Xue-Wei Xu

**Affiliations:** 1 Key Laboratory of Marine Ecosystem and Biogeochemistry, Second Institute of Oceanography, State Oceanic Administration, Hangzhou, 310012 China; 2 College of Food and Biological Engineering, Jiangsu University, Zhenjiang, 212013 China

**Keywords:** Metagenomics library, Esterase, Family IV, Deep-sea, Substrate-binding pocket, Homology modeling

## Abstract

**Background:**

The deep-sea environment harbors a vast pool of novel enzymes. Owing to the limitations of cultivation, cultivation-independent has become an effective method for mining novel enzymes from the environment. Based on a deep-sea sediment metagenomics library, lipolytic-positive clones were obtained by activity-based screening methods.

**Results:**

Two novel esterases, DMWf18-543 and DMWf18-558, were obtained from a deep-sea metagenomic library through activity-based screening and high-throughput sequencing methods. These esterases shared 80.7% amino acid identity with each other and were determined to be new members of bacterial lipolytic enzyme family IV. The two enzymes showed the highest activities toward *p*-nitrophenyl (*p*-NP) butyrate at pH 7.0 and 35–40 °C and were found to be resistant to some metal ions (Ba^2+^, Mg^2+^, and Sr^2+^) and detergents (Triton X-100, Tween 20, and Tween 80). DMWf18-543 and DMWf18-558 exhibited distinct substrate specificities and preferences. DMWf18-543 showed a catalytic range for substrates of C2–C8, whereas DMWf18-558 presented a wider range of C2–C14. Additionally, DMWf18-543 preferred *p*-NP butyrate, whereas DMWf18-558 preferred both *p*-NP butyrate and *p*-NP hexanoate. To investigate the mechanism underlying the phenotypic differences between the esterases, their three-dimensional structures were compared by using homology modeling. The results suggested that residue Leu199 of DMWf18-543 shortens and blocks the substrate-binding pocket. This hypothesis was confirmed by the finding that the DMWf18-558-A199L mutant showed a similar substrate specificity profile to that of DMWf18-543.

**Conclusions:**

This study characterized two novel homologous esterases obtained from a deep-sea sediment metagenomic library. The structural modeling and mutagenesis analysis provided insight into the determinants of their substrate specificity and preference. The characterization and mechanistic analyses of these two novel enzymes should provide a basis for further exploration of their potential biotechnological applications.

**Electronic supplementary material:**

The online version of this article (10.1186/s12934-018-0864-4) contains supplementary material, which is available to authorized users.

## Background

Esterases (EC 3.1.1.1) are lipolytic enzymes that catalyze the cleavage or formation of water-soluble short-chain fatty esters (< 10 carbon chain), whereas lipases (EC 3.1.1.1) prefer water-insoluble long-chain fatty esters (> 10 carbon chain) [1]. A considerable number of microbial esterases have been discovered and studied because of their potential for application in the agriculture, food, pharmaceutical and bioremediation industries [2, 3]. Owing to the limitations of cultivation, activity-based or sequence-based screening of enzymes from metagenomic libraries has become an effective and valuable approach for directly mining esterases from environment samples [4–6].

Bacterial lipolytic enzymes were classified into 17 families (I to XVII) [1, 7, 8]. Among these families, family IV (also known as the HSL family) esterases share remarkable sequence similarity with mammalian hormone-sensitive lipase (HSL) [1], which plays an important role in the regulation of fat cell lipolysis. Recently, numbers of family IV esterases have been isolated from various environments, such as marine environments [9–12], soil [13, 14], activated sludge [15] and the bovine rumen [16], through metagenomic methods.

Pairs of enzymes showing high sequence identity but some phenotypic differences are good materials for studying the mechanisms of catalysis. Their amino acid sequences and crystal structures or structural models can be compared to reveal the sequence or structural differences that cause phenotypic differences. For example, a comparison of the structures of the esterases *Rm*EstA (preferring longer-chain esters) and *Rm*EstB (preferring shorter-chain esters) has revealed that residues Phe222 and Trp92 of *Rm*EstB are related to the shape of the substrate-binding pocket, thereby affecting substrate specificity [17]. Additionally, structural and biochemical analyses of the esterases EstFa_R (pH optimum of 5.0) and SshEstI (pH optimum of 8.0) have been carried out to investigate the mechanism underlying low-pH adaptation, and the unique extended hydrogen bond network around the catalytic triad and the negatively charged surface around the active site have been found to result in a low-pH optimum of EstFa_R [18].

A metagenomic library has previously been constructed from Pacific Ocean deep-sea sediment, from which several lipolytic enzymes have been screened [10]. In the present study, further activity-based screening for lipolytic enzymes was performed; two novel esterase genes (*dmwf18*-*543* and *dmwf18*-*558*) were obtained using high-throughput sequencing method. The encoded enzymes, DMWf18-543 and DMWf18-558, showed high amino acid sequence identities but different enzymatic characteristics compared with esterases from the same metagenomic library previously obtained by Jiang et al. [10]. On the basis of the high sequence identity and the differences between the two enzymes in terms of substrate specificity and preference, their sequences and structural models were compared, and insights into the determinants of their phenotypic differences were obtained.

## Methods

### Metagenome sequencing and analysis

A deep-sea sediment sample (depth of 5886 m) was collected from the skirt of a seamount in the central Pacific Ocean and used to construct a fosmid metagenomic library, as described previously [10]. In this study, *Escherichia coli* EPI300 cells harboring fosmids were spread on Luria–Bertani (LB) agar medium supplemented with 12.5 µg/ml chloramphenicol and 1% tributyrin, and lipolytic-positive clones were screened by using the clear zone method [10]. The LB medium contained 10 g/L NaCl, 10 g/L tryptone, and 5 g/L yeast extract (BD, USA), at pH 7.0. To harvest large amounts of pCC2FOS fosmid DNA for sequencing, the *E. coli* EPI300 cells were cultivated with 2 μl/mL CopyControl™ Fosmid Autoinduction Solution (Epicentre Biotechnologies, USA) for 17 h at 250 rpm. The fosmid DNA was extracted using Axygen Plasmid Miniprep Kit (Corning, USA) and treated with plasmid-safe ATP-dependent DNase (Epicentre Biotechnologies, USA) to remove chromosomal DNA contamination from the host strain.

High-throughput sequencing of the fosmid vectors was carried out on the Illumina HiSeq 2000 platform by Novogene Bioinformatics Technology Co. Ltd. (Beijing). One paired-end (2 × 125 bp) library was constructed, with an approximate insert size of 500 bp. Reads were assembled de novo into contigs by using ABySS version 1.5.2 [19]. Assembly k-values from k = 48 to 64 were tested to identify the optimal value, of k = 62, by using the *abyss*-*pe* script. Vector contamination was filtered out via BLAST searches against the pCC2FOS2™ fosmid sequence (NCBI Accession Number EU140752). Open reading frame (ORF) prediction was performed by using MetaGeneMark [20]. The functional annotation of amino acid sequences of ORFs was performed with the BLASTP program (from the NCBI-Blast-2.2.29 + command-line package) [21] against the GenBank nr [22] and Cluster of Orthologous Groups (COG) [23] databases as well as with the PROKKA package [24].

### Sequence analysis of esterase genes

Two highly related putative esterase genes designated *dmwf18*-*543* and *dmwf18*-*558* were screened, and their deduced amino acid sequences were analyzed using the BLASTP program (https://blast.ncbi.nlm.nih.gov) [25]. Multiple sequence alignment of their amino acid sequences was performed using Clustal X version 2 [26] and ESPript 3.0 [27]. The corresponding phylogenetic tree was constructed using the neighbor-joining method [28] with MEGA version 7.0 software [29].

### Homology modeling and putative structure analysis

The three-dimensional (3D) structures of DMWf18-543 and DMWf18-558 were modeled using the SWISS-MODEL server (https://swissmodel.expasy.org/) [30, 31]. Structural figures were generated using PyMOL software (http://pymol.sourceforge.net).

### Cloning, mutation, expression, and purification

The genes were amplified using PrimeSTAR HS DNA polymerase (TaKaRa, China). The primers 5ʹ-TCGCGGATCCATGGCCAGCCCACAGCT-3ʹ (*Bam*HI site underlined) and 5ʹ-TCCGCTCGAGCTAGCGTGCGGCGGCGG-3ʹ (*Xho*I site underlined) were used to amplify the full-length *dmwf18*-*543* gene; and the primers 5ʹ-TCGCGGATCCATGGCGAGTCCACAGCTCC-3ʹ (*Bam*HI site underlined) and 5ʹ-ATTTGCGGCCGCCTAGCGTGCGGCGGC-3ʹ (*Not*I site underlined) were used to amplify *dmwf18*-*558*. The products were digested by corresponding restriction endonucleases (New England Biolabs, USA) and cloned into pSMT3 expression vector that had been digested with the same enzymes using T4 DNA ligase (New England Biolabs, USA). The recombinant plasmids were then transformed into *E. coli* Rosetta (DE3) cells for protein expression. Transformants harboring the recombinant plasmids were identified via PCR and further confirmed through DNA sequencing. Point mutants were generated by site-directed mutagenesis using the *Fast* Mutagenesis System (Transgene Biotech, China), with wild-type plasmids as templates. The cells were cultivated at 37 °C in LB medium with 50 µg/mL kanamycin and 200 rpm until the optical density (OD_600_) reached approximately 0.6 and were then induced with 0.5 mM of isopropyl-β-d-thiogalactopyranoside (IPTG) at 20 °C and 200 rpm. After cultivation for 16 h, the cells were collected via centrifugation at 12,000 rpm and 4 °C and then washed with phosphate-buffered saline (0.8% NaCl, 0.02% KCl, 0.142% Na_2_HPO_4_, 0.027% KH_2_PO_4_, pH 7.4). Next, the cells were suspended in 20 mM imidazole buffer (500 mM NaCl, 20 mM Tris–HCl, pH 8.0) and subjected to ultrasonic disruption (350 W). After centrifugation at 12,000 rpm and 4 °C for 30 min, the supernatant was purified with Ni Sepharose (GE, USA) to obtain the N-terminal His-tagged small ubiquitin-related modifier (SUMO) fusion. The fusion protein was cleaved with ubiquitin-like specific protease 1 (ULP1) with overnight dialysis at 4 °C. Subsequently, the products were passed through the Ni Sepharose column again to capture the His-tagged SUMO. The recombinant protein in the eluate was verified via sodium dodecyl sulfate polyacrylamide gel electrophoresis (SDS-PAGE) using 12% polyacrylamide gels.

### Enzyme activity assay

Esterase activity assays were performed using a spectrophotometric method. The standard reaction mixture contained 10 µL of 100 mM *p*-NP butyrate, 980 µL of phosphate buffer (100 mM, pH 7.5), and the purified enzyme in a final volume of 1 mL. The activity of the enzyme was quantified at 35 °C, and the release of *p*-nitrophenol was measured at 405 nm over 2 min with a DU800 ultraviolet–visible spectrophotometer (Beckman, USA). All values were determined in triplicate, and reactions with the added thermally inactivated enzyme were used as controls. One unit of enzyme activity was defined as the amount of esterase required to release 1 µmol of *p*-nitrophenol per minute from the *p*-NP ester.

### Enzyme characterization

The kinetic parameters were obtained using *p*-NP butyrate and *p*-NP hexanoate as substrates at various concentrations (0.05, 0.1, 0.2, 0.4, 0.6, 0.8, 1.0, 1.2, 1.5, 1.8, and 2.0 mM). The kinetic parameters were calculated by analyzing the slopes of the Michaelis–Menten equation by using GraphPad software (GraphPad Inc., USA).

To determine substrate specificity, *p*-NP esters with various acyl chain lengths (C2–C16) were added to the reaction mixture at a final concentration of 1 mM. The substrates used in the study were *p*-nitrophenyl (*p*-NP) acetate (C2), *p*-NP butyrate (C4), *p*-NP caprylate (C8), *p*-NP decanoate (C10), *p*-NP laurate (C12), *p*-NP myristate (C14), and *p*-NP palmitate (C16) from Sigma-Aldrich (USA), and *p*-NP hexanoate (C6) from TCI (Japan).

The optimum temperature for enzyme activity was assessed over a range of 15–60 °C at intervals of 5 °C. The optimum pH for enzyme activity was determined over a pH range from 3.0 to 10.0. The release of *p*-nitrophenol at different pH values was measured at 348 nm, the pH-independent isosbestic wavelength of *p*-nitrophenol and *p*-nitrophenolate. The buffers used were 100 mM citrate buffer (pH 3.0–6.0), 100 mM phosphate buffer (pH 6.0–7.5), 100 mM tricine buffer (pH 7.5–9.0), and 50 mM CHES buffer (pH 9.0–10.0).

The effect of the chelating agent ethylenediaminetetraacetic acid (EDTA) was evaluated at a final concentration of 10 mM. The effects of various metal ions (Ba^2+^, Ca^2+^, Co^2+^, Cu^2+^, Mg^2+^, Mn^2+^, Ni^2+^, Sr^2+^ and Zn^2+^) were examined at a final concentration of 10 mM. The effects of various detergents, including SDS, Triton X-100, Tween-20, and Tween-80, were determined at a final concentration of 1% (v/v, except w/v for SDS). The effects of various organic solvents, including acetone, acetonitrile, ethanol, *N*,*N*-dimethylformamide (DMF), dimethyl sulfoxide (DMSO), glycerol, isopropanol, and methanol, were measured at final concentrations of 5 and 15% (v/v).

All values were determined in triplicate. Data were presented as mean ± SD. Statistical analyses were performed with two-tailed unpaired Student’s *t*-tests. P values less than 0.05 were considered statistically significant.

### Nucleotide sequence accession numbers

The nucleotide sequences of contigs S36 and S37 have been deposited into the GenBank database under Accession Numbers MF770983 and MF770984. The locus_tags of *dmwf18*-*543* and *dmwf18*-*558* esterase genes are S36_0543 and S37_0558.

## Results

### Sequence analysis of fosmid clones

Eighteen lipolytic-positive clones were screened through activity-based screening and identified on the basis of clear zones around the colonies on LB agar medium supplemented with 1% tributyrin [10]. The fosmid clones were then mixed and used in high-throughput sequencing. In total, 40 contigs were assembled and annotated (data not shown). Among these contigs, S36 and S37 were 11,479 bp and 11,464 bp long and showed high sequence similarity with each other (97.3%, 11,176/11,488). Both of the contigs contained 14 ORFs with the same gene arrangement (Additional file 1: Table S1). The ORFs of the two contigs exhibited high sequence identities: 72.2–100.0% for their nucleotide sequences and 83.7–100.0% for their deduced amino acid sequences (Additional file 1: Table S1). The deduced amino acid sequences indicated that 8 out of the 14 ORFs could be assigned by using the COG classification system. Of these 8 ORFs, three encoded functions related to energy production and conversion, two related to lipid transport and metabolism, and one related to replication, recombination and repair.

### Sequence analysis of the novel esterases

Two putative lipolytic enzyme genes of 909 bp were identified, encoding proteins of 302 amino acids; these genes were assigned as lipid transport and metabolism genes according to COG categories and designated *dmwf18*-*543* and *dmwf18*-*558*, respectively (Additional file 1: Table S1). These proteins shared 80.7% (234/290) amino acid sequence identity with each other. Comparison of the amino acid sequences with known esterases in the GenBank nr database showed that DMWf18-543 and DMWf18-558 were similar (> 50% identities) to several esterases from metagenomes (Table 1). DMWf18-543 and DMWf18-558 shared 81.4% and 82.4% sequence identity (246/302 and 249/302) with esterase Est6 and 55.0 and 57.0% identity with Est2 or Est4 identified previously from the same metagenome (GenBank Accession No. AFB82690, AFB82678, and AFB82687) [10]; 54.0–57.5% identity with EstMY and EstMY09-02 from an activated sludge metagenome (ADM67447 and ADM67446) [15]; and 50.5–52.6% identity with esterases from soil metagenomes (AGF91877, AFC77925, AAS77236, AAS77233, and AAS77238) [32–34].

**Table 1:** Comparison of DMWf18-543 and DMWf18-558 with similar relatives

Enzyme name	Accession no.	Source	Description	Identity (%)		References
				DMWf18-543	DMWf18-558	
Est6	AFB82690	Deep-sea sediment metagenome	Esterase	81.4 (246/302)	82.4 (249/302)	[10]
Est4	AFB82689		Esterase	55.0 (166/302)	57.0 (172/302)	
Est2	AFB82687		Esterase	55.0 (166/302)	57.0 (172/302)	
EstMY	ADM67447	Activated sludge metagenome	Esterase	57.5 (173/301)	57.5 (173/301)	[15]
EstMY09-2	ADM67446		Putative lipolytic protein	57.0 (166/291)	54.0 (157/291)	
ArmEst1	AGF91877	Soil metagenome	Esterase	50.8 (150/295)	51.9 (153/295)	[34]
EstC23	AFC77925	Soil metagenome	Esterase	50.5 (149/295)	51.5 (152/295)	[32]
ELP11B	AAS77236	Soil metagenome	Esterase	51.8 (147/284)	52.5 (149/284)	[33]
ELP45	AAS77233		Esterase	51.8 (147/284)	52.1 (148/284)	
ELP141	AAS77238		Esterase	51.8 (146/282)	52.6 (150/285)	

**Fig. 1:**
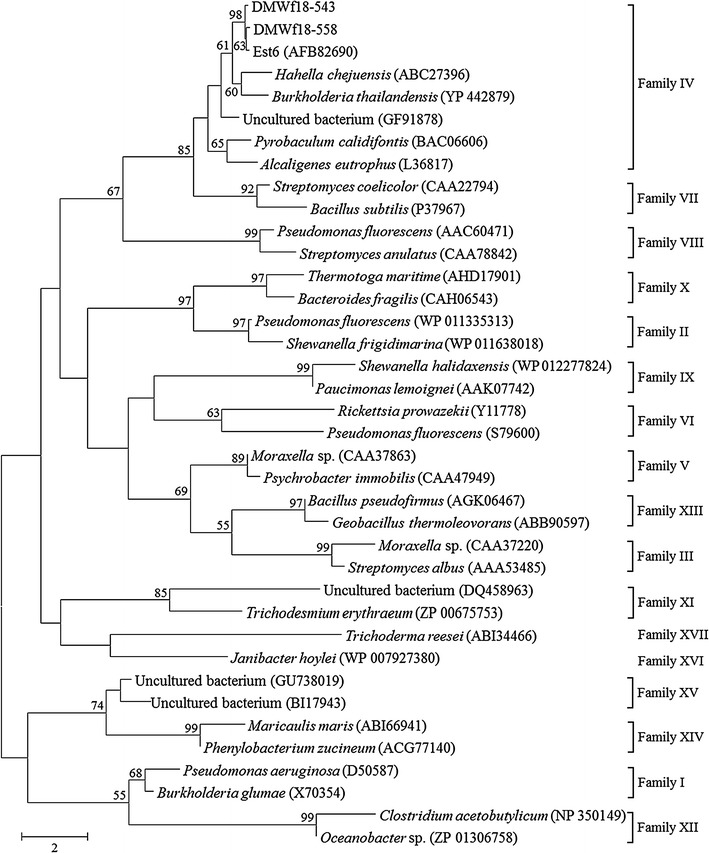
Neighbor-joining phylogenetic tree based on the amino acid sequences of DMWf18-543, DMWf18-558 and related lipolytic enzymes. Sequence alignment was performed using ClustalX, and the tree was constructed using MEGA software. Bootstrap values are based on 1000 replicates, and only values > 50% are shown. The scale bar indicates the number of amino acid substitutions per site

**Fig. 2:**
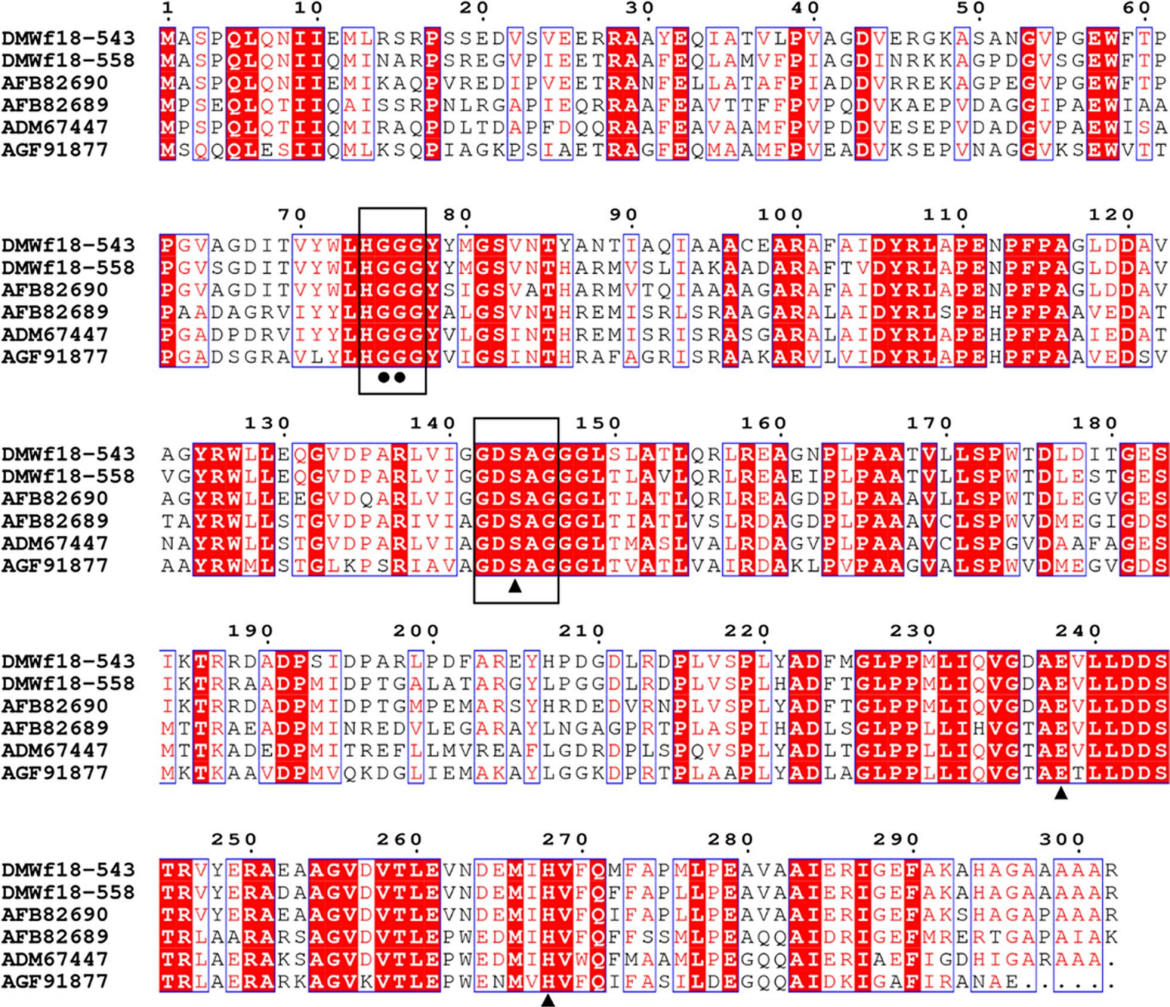
Amino acid sequence alignment of DMWf18-543- and DMWf18-558-related lipolytic enzymes. The accession numbers of the enzymes in the GenBank database are given for DMWf18-543 and DMWf18-558 (from this study), Est6 (AFB82690), Est4 (AFB82689), EstMY (ADM67447), and ArmEst1 (AGF91877). Sequence alignment was performed using the ClustalX and ESPript programs. Identical and similar residues among groups are indicated in white text on a red background and in red text on a white background, respectively. Solid circles indicate the locations of the residues involved in the oxyanion hole (glycine (G)). The triangles indicate the locations of the catalytic active site residues (serine (S), aspartate (D), and histidine (H)). The square indicates the location of residue Leu199 of DMWf18-543 and residue Ala199 of DMWf18-558. The conserved HGGG and GXSXG motifs, in which the oxyanion hole and catalytic triad are located, are outlined with boxes

Phylogenetic analysis was carried out to reveal the relationships between the two esterases and other known bacterial lipolytic enzymes. A phylogenetic tree constructed with 17 bacterial lipolytic enzyme families showed that DMWf18-543 and DMWf18-558 belong to family IV (Fig. 1). Multiple sequence alignment of DMWf18-543 and DMWf18-558 with highly related homologs indicated that their catalytic triad is composed of residues Ser144, Glu238, and His268 (Fig. 2). The catalytic residue Ser144 and the oxyanion hole residues Gly75 and Gly76 are located in the GXSXG (GDSAG, position 142–146) and HGGG (positions 74–77) motifs, which are conserved in the family IV lipolytic enzymes.

### Expression and characterization

The gene fragments encoding the enzymes were cloned into the pSMT3 vector and expressed in *E. coli* Rosetta (DE3). After induction of expression with IPTG at 20 °C for 12 h, the recombinant proteins were purified using His-tag affinity chromatography. The calculated molecular weights (MW) of DMWf18-543 and DMWf18-558 were 32.32 and 32.12 kDa. SDS-PAGE analysis of the purified proteins revealed an approximate MW of 32 kDa, which was consistent with the calculated value of DMWf18-543 and DMWf18-558 (Additional file 2: Figure S1).

The kinetic parameters of purified DMWf18-543 and DMWf18-558 were determined by using *p*-NP butyrate and *p*-NP hexanoate as substrates, and the results are shown in Table 2. When *p*-NP butyrate was used as a substrate, the *V*_max_ and *K*_m_ of DMWf18-543 and DMWf18-558 were 5957 ± 112 µmol/mg/min and 0.33 ± 0.02 mM and 5523 ± 228 µmol/mg/min and 0.60 ± 0.06 mM, respectively.

**Table 2:** Kinetic parameters of DMWf18-543, DMWf18-558 and the mutant protein

Enzyme	pH	*V*max (μmol/mg/min)	*K*_m_ (mM)	*k*cat (s^−1^)	*k*cat/*K*_m_ (mM^−1^/s^−1^)
DMWf18-543	*p*-NP butyrate	5957 ± 112	0.33 ± 0.02	3209 ± 60	9610
	*p*-NP hexanoate	108 ± 3	0.54 ± 0.04	58 ± 1	107
DMWf18-558	*p*-NP butyrate	5523 ± 228	0.60 ± 0.06	2957 ± 122	4854
	*p*-NP hexanoate	5240 ± 145	0.076 ± 0.009	2805 ± 78	37,120
DMWf18-558-A199L	*p*-NP butyrate	2101 ± 32	0.16 ± 0.01	1126 ± 17	7239
	*p*-NP hexanoate	909 ± 14	0.034 ± 0.003	487 ± 8	14,542

**Fig. 3:**
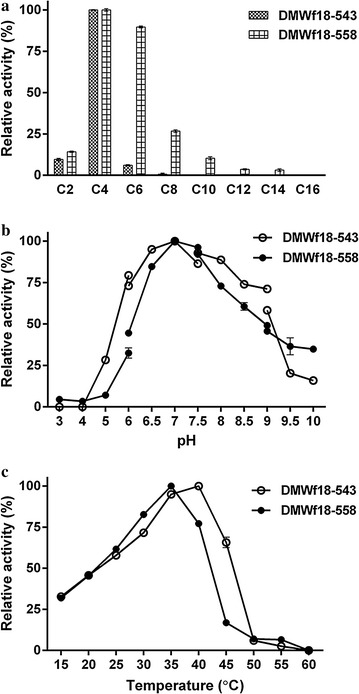
Characterization of DMWf18-543 and DMWf18-558. **a** Substrate specificity was determined using the *p*-NP esters, including *p*-NP acetate (C2), *p*-NP butyrate (C4), *p*-NP caprylate (C8), *p*-NP decanoate (C10), *p*-NP laurate (C12), *p*-NP myristate (C14), and *p*-NP palmitate (C16). All of the tests were performed at 35 °C and pH 7.5. **b** Effects of pH on the activity were determined in different buffers: 100 mM citrate buffer (pH 3.0–6.0), 100 mM phosphate buffer (pH 6.0–7.5), 100 mM tricine buffer (pH 7.5–9.0), and 50 mM CHES buffer (pH 9.0–10.0). All of the tests were performed at 35 °C using *p*-NP butyrate as the substrate. **c** Effects of temperature on the activity were determined at various temperatures at pH 7.5 using *p*-NP butyrate as the substrate. The highest activity was taken as 100%. Data are presented as the mean ± SD (*n* = 3)

The substrate specificities of the enzymes were determined by using *p*-NP esters with various acyl chain lengths (C2–C16) (Fig. 3a). Among the esters tested, DMWf18-543 exhibited the highest activity toward *p*-NP butyrate (4757 U/mg) and only weak activity toward *p*-NP acetate, *p*-NP hexanoate, and *p*-NP caprylate (10, 6, and 1% relative activities). Additionally, no activity was detected toward *p*-NP esters with side chains longer than C10. Nevertheless, DMWf18-558 exhibited high activity toward *p*-NP butyrate (4520 U/mg) and *p*-NP hexanoate (4054 U/mg). DMWf18-558 also retained over 14% and 27% relative activity toward *p*-NP acetate and *p*-NP caprylate, respectively, in addition to showing weak activity toward *p*-NP decanoate, *p*-NP laurate, and *p*-NP myristate (10, 4, and 3%) (Fig. 3a). The optimum activities of the enzymes were measured over a pH range of 3.0–10.0 and a temperature range of 15–60 °C, by using *p*-NP butyrate as the substrate. DMWf18-543 showed the highest activity at pH 7.0 and 35–40 °C and DMWf18-558 at pH 7.0 and 35 °C (Fig. 3b, c).

DMWf18-543 and DMWf18-558 were highly resistant to 10 mM Ba^2+^, Mg^2+^, or Sr^2+^, retaining over 80% of their initial activity. However, their activities were strongly or completely inhibited by Cu^2+^, Ni^2+^, and Zn^2+^ (Table 3). The addition of all tested solvents at 5 and 15% (v/v) inhibited the activities of DMWf18-543 and DMWf18-558, except for glycerol, and over 85% of the initial activity of these enzymes was retained (Table 4). The addition of 1% SDS (w/v) inactivated DMWf18-543 and DMWf18-558. Furthermore, the activity of DMWf18-543 decreased to 45–67% when 1% Triton X-100, Tween 20, or Tween 80 (v/v) was added, whereas the activity of DMWf18-558 decreased to 10–29% when the detergents were added at 1% (Table 4).

**Table 3:** Effects of various metal ions and chelation on the activity of DMWf18-543 and DMWf18-558

Metals and chelating agent/10 mM	Relative activity (%)	
	DMWf18-543	DMWf18-558
Control	100.0 ± 0.5	100.0 ± 2.0
Ba*	88.0 ± 0.2	97.9 ± 0.3
Ca*	78.1 ± 0.8	81.8 ± 2.0
Co*	30.9 ± 0.1	15.6 ± 1.3
Cu	0	0
Mg	97.8 ± 0.4	92.6 ± 5.9
Mn*	38.4 ± 0.4	35.2 ± 1.1
Ni*	22.4 ± 1.8	0
Sr*	80.4 ± 0.6	99.8 ± 0.9
Zn*	21.2 ± 0.4	15.9 ± 3.1
EDTA	117.7 ± 0.5	124.9 ± 4.3

The activity observed without metal ion or chelation was taken as 100%. * *p* < 0.05, representing a significant difference between DMWf18-543 and DMWf18-558 (Student’s *t* test)

**Table 4:** Effects of various organic solvents and detergents on the activity of DMWf18-543 and DMWf18-558

Solvents/5%	Relative activity (%)		Solvents/15%	Relative activity (%)		Detergents/1%	Relative activity (%)	
	DMWf18-543	DMWf18-558		DMWf18-543	DMWf18-558		DMWf18-543	DMWf18-558
Control	100.0 ± 0.7	100.0 ± 1.0	Control	100.0 ± 1.2	100.0 ± 2.5	Control	100 ± 1.1	100 ± 0.3
Acetone*	57.8 ± 0.1	20.8 ± 0.4	Acetone	0	0	SDS	0	0
Acetonitrile*	53.0 ± 0.1	33.8 ± 0.3	Acetonitrile	0	0	TritonX-100*	45.1 ± 0.9	9.5 ± 0.1
Alcohol*	56.4 ± 0.4	31.2 ± 0.3	Alcohol*	0	4.0 ± 0.7	Tween 20*	66.6 ± 0.5	28.6 ± 0.9
DMF*	58.6 ± 0.7	14.0 ± 0.6	DMF	0	0	Tween 80*	56.1 ± 0.6	11.3 ± 0.6
DMSO*	90.4 ± 0.1	52.5 ± 2.3	DMSO*	38.0 ± 0.1	23.8 ± 0.3			
Glycerol	98.0 ± 0.8	98.2 ± 2.4	Glycerol*	85.2 ± 0.7	103.0 ± 1.9			
Isopropanol*	67.1 ± 0.6	20.7 ± 0.1	Isopropanol	0	0			
Methanol*	81.0 ± 0.3	75.6 ± 1.9	Methanol*	5.5 ± 0.1	0			

The activity observed without an organic solvent or detergent was taken as 100%. * *p* < 0.05, representing a significant difference between DMWf18-543 and DMWf18-558 (Student’s *t* test)

### Overall structural models

**Fig. 4:**
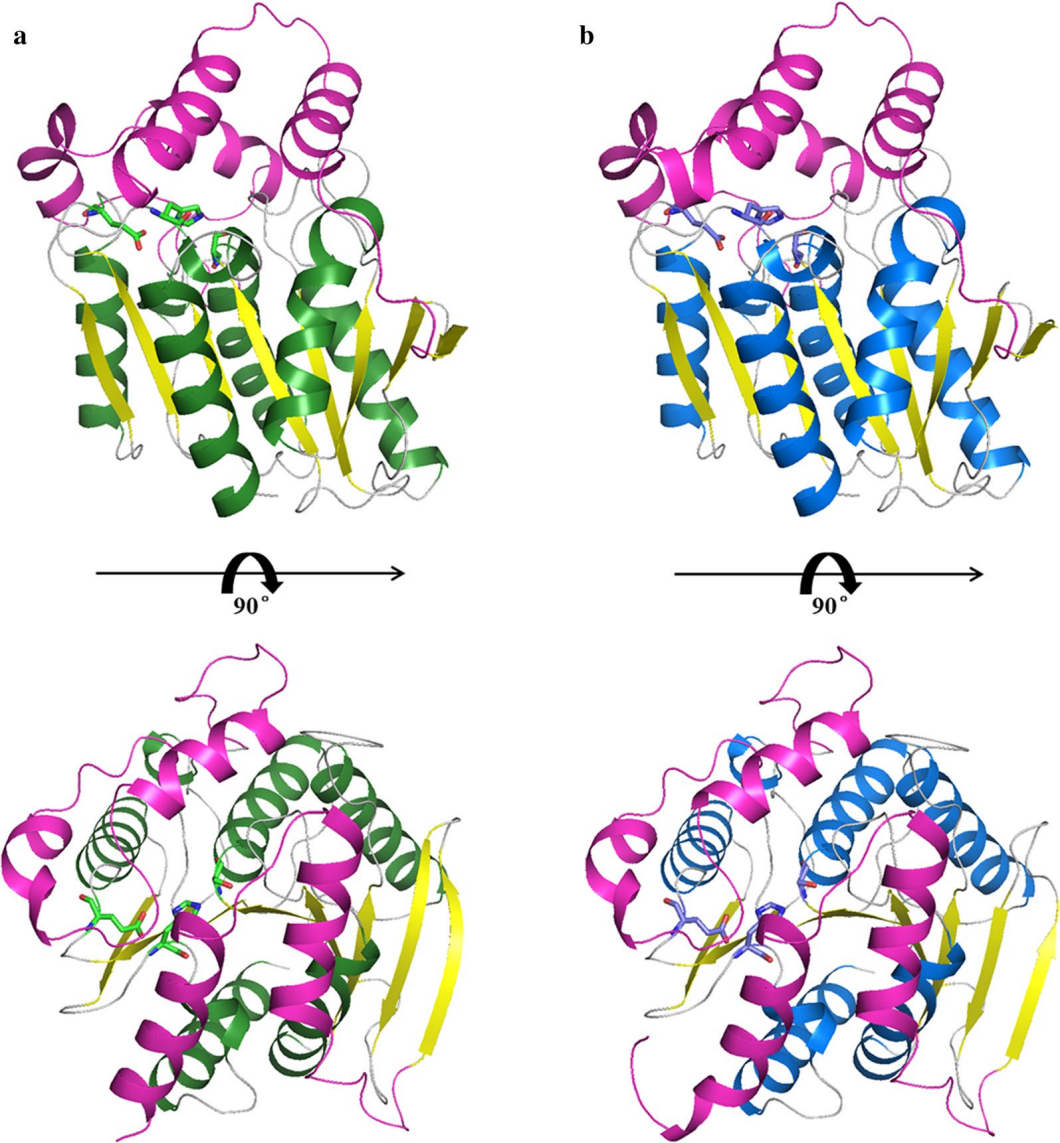
Cartoon representation of 3D structural models of DMWf18-543 (**a**) and DMWf18-558 (**b**). The catalytic domains are shown in green/blue and yellow, and the cap domains are shown in magenta. The catalytic triad residues are indicated as stick models

The 3D structures of DMWf18-543 and DMWf18-558 were modeled using the SWISS-MODEL server. Both structures could be divided into two domains: a catalytic domain (residues 45–175 and 217–295 for DMWf18-543 and DMWf18-558) with a canonical α/β-hydrolase fold consisting of eight parallel β strands surrounded by five α helices and a cap domain (residues 4–44 and 176–216 for DMWf18-543, residues 1–44 and 176–216 for DMWf18-558) (Fig. 4).

### Comparison of substrate-binding pockets

**Fig. 5:**
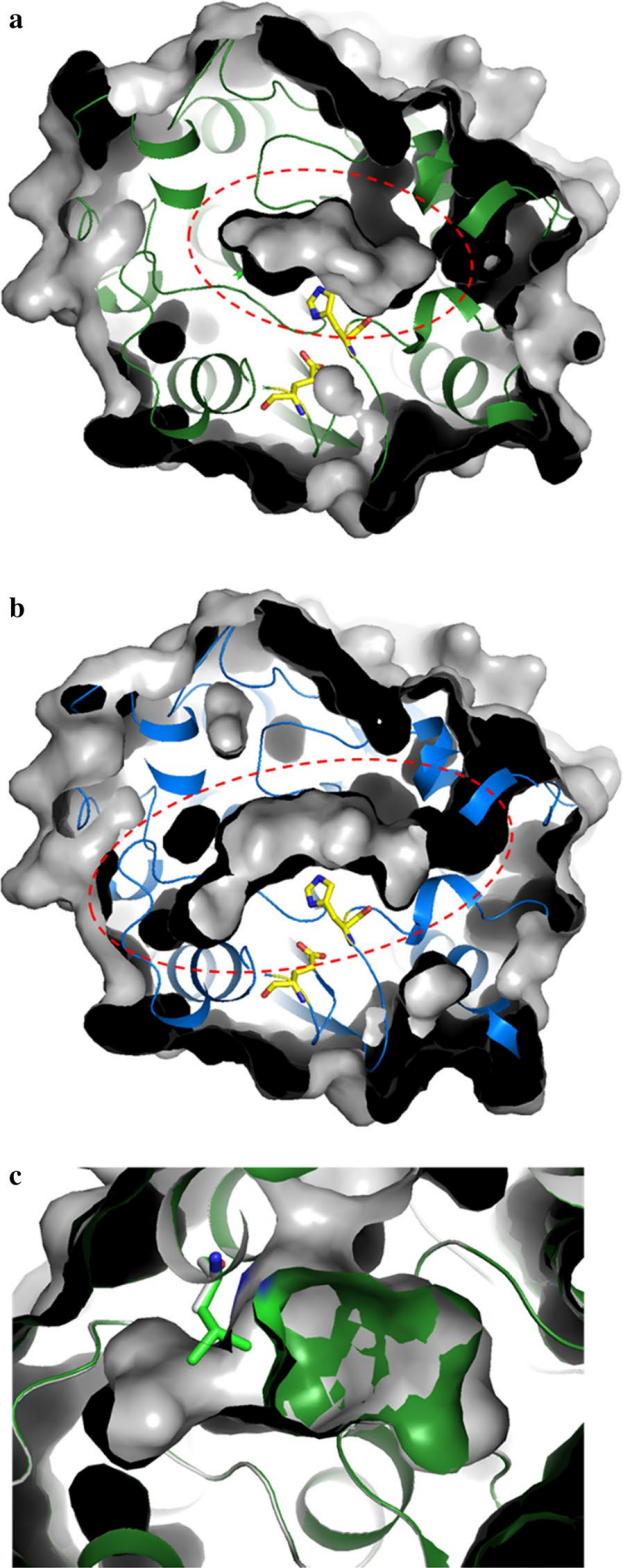
Substrate-binding pockets of DMWf18-543 and DMWf18-558. Surface view of the structures showing the differences in the substrate-binding pockets of DMWf18-543 (**a**) and DMWf18-558 (**b**). The substrate-binding pockets are indicated with red dashed lines. Residues of the catalytic triad are shown as stick models in yellow. **c** Superposition of the surfaces of DMWf18-543 (green) and DMWf18-558 (gray). Leu199 of DMWf18-543 and Ala199 of DMWf18-558 are shown as stick models

Superposition of the surfaces of DMWf18-543 and DMWf18-558 showed differences in their substrate-binding pockets (Fig. 5). The substrate-binding pocket of DMWf18-543 exhibited a short channel, whereas that of DMWf18-558 had a longer channel (~ 8 Å longer) (Fig. 5a, b). The difference in the shape of the substrate-binding pockets of the two esterases results in differences in the favored carbon chain lengths of the ester substrates (Fig. 3a). Superposition of the surface of DMWf18-558 and sticks of DMWf18-543 showed that residue Leu199 of DMWf18-543 might shorten and block the substrate-binding pocket (Fig. 5c), thus potentially leading to little DMWf18-543 activity toward *p*-NP esters with side chains longer than C6. Additionally, DMWf18-558, with relatively high activity toward *p*-NP hexanoate and *p*-NP caprylate, exhibited the relatively small side-chain residue Ala199 at the same location.

**Fig. 6:**
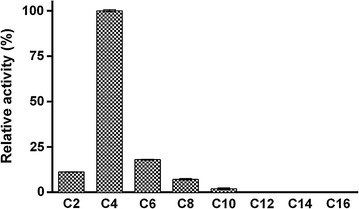
Substrate specificity of DMWf18-558-A199L. The esterase activity of the mutant was determined using *p*-NP esters. The highest activity was taken as 100%. Data are presented as the mean ± SD (*n* = 3)

To verify the above hypothesis, two mutants (DMWf18-543-L199A and DMWf18-558-A199L) were designed, and only DMWf18-558-A199L was successfully expressed. The substrate specificity of the mutant was determined. Compared with the wild-type enzymes, the activity of the DMWf18-558-A199L mutant toward *p*-NP hexanoate and *p*-NP caprylate decreased markedly to 18 and 7% (Fig. 6). Additionally, little activity was detected toward *p*-NP esters with side chains longer than C10 (Fig. 6). The results suggested that residue 199 is the determinant of substrate specificity and preference.

## Discussion

In this study, we identified two novel esterase genes through activity-based screening and high-throughput fosmid sequencing from a metagenomic library from Pacific Ocean deep-sea sediment. We analyzed and compared the biochemical characteristics and structural models of the two esterases.

Forty contigs were obtained after 18 lipolytic-positive fosmid clones were sequenced and assembled. The two novel esterase genes were located on two contigs showing high sequence similarity and the same gene arrangement. Each contig contained 14 ORFs, and half of the ORFs (7 out of 14) presented the most significant BLAST hits to members of *Chloroflexi* (Additional file 1: Table S1), thus indicating that the two genome fragments might belong to uncultured *Chloroflexi* bacteria. *Chloroflexi* are quite frequently found in deep-sea surface sediment environments [35–37]. Additionally, a *Chloroflexi* genome fragment has previously been found and sequenced in the same metagenomic library [38].

The two novel esterases, DMWf18-543 and DMWf18-558, shared high amino acid identity with each other (80.7%) and showed the highest sequence identity to Est6, Est2 and Est4 among members of the database (55.0–82.4%), all of which were identified from the same metagenomic library through function-driven screening and subcloning [10]. The enzymatic characteristics of these esterases, which exhibited the same origin and shared high sequence identity, were compared. Est6 is a cold-activated esterase and exhibited its highest activity at 20 °C [10], whereas Est2 and Est4 exhibited their highest activity at higher temperatures (40–45 °C, data not shown), and DMWf18-543 and DMWf18-558 showed their highest activity at 35–40 °C. A comparison of DMWf18-543 with DMWf18-558 indicated that they displayed similar temperature and pH optimums, and the differences in the effects of various organic solvents, detergents and metals between them were not very large. However, in terms of their activity toward *p*-NP esters with different side-chain lengths, DMWf18-543 and DMWf18-558 showed notable differences. DMWf18-543 exhibited the highest activity toward *p*-NP butyrate and little activity toward *p*-NP hexanoate or *p*-NP esters with longer side chains. However, DMWf18-558 preferred both *p*-NP butyrate and *p*-NP hexanoate (about 90%) and retained 27% activity toward *p*-NP caprylate and even some activity toward *p*-NP esters with side chains of C10–C14.

To investigate the structural basis of the different substrate specificities and preferences of the enzymes, we attempted to obtain their crystal structures. Unfortunately, crystals did not appear and grow (data not shown). Thus, structural models of DMWf18-543 and DMWf18-558 were constructed and compared. The model comparison results suggested that DMWf18-543 has a short substrate-binding channel, whereas DMWf18-558 has a relatively long substrate-binding channel (Fig. 5a, b). The substrate-binding channel of DMWf18-543 was shortened by residue Leu199 (Fig. 5c), which might result in low or no activity toward *p*-NP esters with longer side chains. Residues Leu199 of DMWf18-543 and Ala199 of DMWf18-558 were selected for site-directed mutagenesis to investigate their roles in the substrate accommodation of substrate-binding pockets. Finally, DMWf18-558-A199L was induced and expressed successfully. The substitution of leucine for alanine caused the substrate-binding pocket to shorten, and the substrate specificity profile of DMWf18-558-A199L was similar to that of DMWf18-543 (Figs. 3a, 6). Furthermore, the DMWf18-558-A199L mutant retained little activity toward *p*-NP esters with side chains longer than C6.

The substrate specificity or preference of esterases toward esters with different acyl chain lengths is usually attributed to the shape of the substrate-binding pocket. A previous comparison of the crystal structures of two family IV esterases (*Rm*EstA and *Rm*EstB) from *Rhizomucor miehei* [17] has shown that two aromatic amino acids (Phe222 and Trp92) in the substrate-binding pocket of *Rm*EstB narrow its substrate-binding pocket as well as its substrate specificity. Phe222 and Trp92 of *Rm*EstB correspond to Tyr78 and Ala203 of DMWf18-543 and DMWf18-558, which are not located in their substrate-binding pockets. Thus, these two residues were not considered to be determinants of substrate specificity or preference on the basis of present study.

The mutation of residue Ala199 also affected the catalytic activity and substrate affinity of the enzyme. A decrease in the *K*_m_ value and an increase in the *k*cat value toward *p*-NP butyrate were observed (from 0.60 ± 0.06 mM and 2957 ± 122/s to 0.16 ± 0.01 mM and 1126 ± 17/s, respectively), thus suggesting that the ability to bind substrate was enhanced and that the turnover rate of the enzyme–substrate complex to product and enzyme increased. Therefore, the catalytic efficiency of DMWf18-558-A199L against *p*-NP butyrate was higher than that of wild-type DMWf18-558, as indicated by a *k*cat/*K*_m_ value of 7239/mM/s (Table 2).

The stronger substrate affinity and higher catalytic efficiency of DMWf18-558-A199L might make it more advantageous in industrial applications. In addition, the metal ion and detergent tolerant properties make DMWf18-543 and DMWf18-558 good candidates for biocatalytic processes requiring or containing metal ions or detergents, such as laundry detergent, textile, bioremediation and waste treatment.

In conclusion, two novel esterase genes with high similarity were screened, cloned and expressed from a deep-sea sediment metagenomic library. The enzymes were characterized and showed different hydrolysis abilities toward esters with different acyl chain lengths. Additionally, the structural modeling and mutagenesis of DMWf18-543 and DMWf18-558 provided insight into the determinants of their substrate specificity and preference. The characterization and mechanistic analysis of these enzymes should provide a basis for further exploration of their potential biotechnological applications.

## Additional files

**Additional file 1: Table S1.** Predicted protiens encoded by the fosmid clones.

**Additional file 2: Figure S1.** SDS-PAGE of purified DMWf18-543 and DMWf18-558. Lane 1, purified DMWf18-543; Lane 2, purified DMWf18-558; Lane M, marker.

## Abbreviations

3D: three-dimension
COG: Cluster of Orthologous Groups
DMF: *N*,*N*-dimethylformamide
DMSO: dimethyl sulfoxide
EDTA: ethylenediaminetetraacetic acid
HSL: hormone-sensitive lipase
IPTG: isopropyl-β-d-thiogalactopyranoside
LB: Luria–Bertani
*p*-NP: *p*-nitrophenyl
ORF: open reading frame
SDS-PAGE: sodium dodecyl sulfate polyacrylamide gel electrophoresis
