# Supplementary material for: Two novel deep-sea sediment metagenome-derived esterases: residue 199 is the determinant of substrate specificity and preference
Source: Microb Cell Fact. 2018 Jan 30;17:16. doi: 10.1186/s12934-018-0864-4 (PMC5789746; doi:10.1186/s12934-018-0864-4)

**Additional file 2: Figure S1.** SDS-PAGE of purified DMWf18-543 and DMWf18-558. Lane 1, purified DMWf18-543; Lane 2, purified DMWf18-558; Lane M, marker.


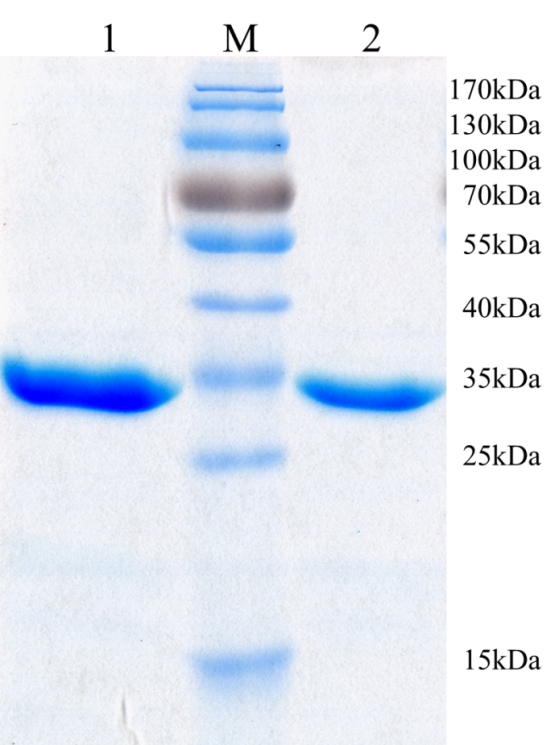

Supplement: Supplementary file 2 — Additional file 2: Figure S1. SDS-PAGE of purified DMWf18-543 and DMWf18-558. Lane 1, purified DMWf18-543; Lane 2, purified DMWf18-558; Lane M, marker. [file 12934_2018_864_MOESM2_ESM.docx]
